# Association of LAMA1 Single-Nucleotide Polymorphisms with Risk of Esophageal Squamous Cell Carcinoma among the Eastern Chinese Population

**DOI:** 10.1155/2023/6922909

**Published:** 2023-02-14

**Authors:** Shaoyuan Zhang, Yong Fang, Feng Su, Tian Jiang, Jinjie Yu, Siyun Lin, Lu Lv, Tao Long, Huiwen Pan, Junqing Qi, Qiang Zhou, Weifeng Tang, Guowen Ding, Liming Wang, Lijie Tan, Jun Yin

**Affiliations:** ^1^Department of Thoracic Surgery, Zhongshan Hospital, Fudan University, Shanghai 200032, China; ^2^Cancer Center, Zhongshan Hospital, Fudan University, Shanghai 200032, China; ^3^Department of Cardiothoracic Surgery, Affiliated People's Hospital of Jiangsu University, Jiangsu 212002, China; ^4^Department of Thoracic Surgery, Sichuan Cancer Hospital and Institute, Sichuan 610042, China; ^5^Department of Cardiothoracic Surgery, Nanjing Drum Tower Hospital, Jiangsu 210008, China; ^6^Department of Respiratory, Shanghai Xuhui Central Hospital, Shanghai 200031, China

## Abstract

**Introduction:**

LAMA1, also known as laminin subunit *α*1, is a member of the laminin family, which is widely reported to be a key basement membrane molecule that affects various biological activities and is associated with many kinds of diseases. We aimed to investigate the association between *LAMA1*single-nucleotide polymorphisms and the occurrence and progression of esophageal squamous cell carcinoma in the Chinese population.

**Method:**

2,186 participants were collected retrospectively between October 2008 and January 2017, including 1,043 ESCC patients and 1,143 noncancer patients. A 2 mL blood sample was obtained intravenously for the LDR for SNP analysis. The 6 SNP loci of *LAMA1* were selected and examined. We analyzed the association of several genetic models of 6 *LAMA1* SNP loci, sex, age, smoking and drinking status, and the occurrence of esophageal squamous cell carcinoma.

**Results:**

In the rs62081531 G > A locus, genotype GA was a protective factor for ESCC compared with GG (OR: 0.830, *P*=0.046), especially among the younger and nondrinkers. At rs607230 T > C, genotype TC was linked with a lower risk of ESCC compared with TT. (OR: 0.613, *P*=0.034). Haplotype Frequencies revealed that A_rs62081531_G_rs621993_A_rs539713_T_rs566655_A_rs73938538_C_rs607230_ (OR: 0.803, *P*=0.028) and G_rs62081531_G_rs621993_A_rs539713_T_rs566655_C_rs73938538_C_rs607230_ (OR: 0.679, *P*=0.010) were strongly associated with lower susceptibility of ESCC.

**Conclusion:**

The *LAMA1* rs62081531, rs539713, rs566655, and rs607230 polymorphisms were demonstrated to be related to susceptibility to ESCC in the Chinese population. *LAMA1* SNPs may have a significant impact on the occurrence of esophageal cancer and may serve as potential diagnostic biomarkers.

## 1. Introduction

Esophageal cancer is a prevalent malignant tumor that has a high rate of morbidity and mortality worldwide. According to the Global Cancer Statistics 2020, esophageal cancer is more prevalent in East Asia, particularly in China, as well as West Asia and Africa [1]. There was a clear correlation between the pathological type of esophageal cancer and its geographic distribution. Squamous cell carcinoma is the most common type of esophageal cancer in developing countries. China has a high prevalence of esophageal squamous cell carcinoma (ESCC) of up to 90%. A variety of pathogenic factors may lead to esophageal squamous cell carcinoma, including smoking, drinking, eating habits, and viral infections [2, 3]. However, not everyone exposed to these risk factors develops esophageal cancer, suggesting that genetic susceptibility, particularly single nucleotide polymorphisms (SNPs), plays a significant role in the development of esophageal squamous cell carcinoma.

LAMA1 is also known as laminin subunit *α*1. Laminins are a family of glycoproteins found in the extracellular matrix that comprises the basement membrane [4, 5]. Laminins have a heterotrimeric structure composed of an *α*, *β,* and *γ* chain [6]. Numerous biological processes are known to be directed by them, including cell adhesion, mitogenesis, differentiation, and metastasis, all of which contribute to carcinogenesis [7–9]. Tissue distribution of LAMA1 occurred mainly in early epithelial development and some adult epithelia. Recent reports have shown that laminin-1 acts as an efficient attachment protein for a large variety of cultured cell types *in vitro* [10]. Mutations in the *LAMA1* gene result in a deficiency of the laminin *α*1 chain, which may lead to tumorigenesis and progression [11]. Recent research indicates that *LAMA1* mutations or overexpression are linked with the occurrence and development of various malignant tumors, including colon cancer, pancreatic cancer, and ovarian cancer [12–15].

However, the relationship between the *LAMA1*single-nucleotide polymorphism and ESCC remains unclear. Through multicenter large-sample case-control research, we aim to thoroughly investigate the association between *LAMA1* single-nucleotide polymorphisms and the incidence and progression of esophageal cancer.

## 2. Method

### 2.1. Patients and Study Design

Between October 2008 and January 2017, 2,186 participants were collected from the Affiliated People's Hospital and the Affiliated Hospital of Jiangsu University (Zhenjiang, China). Totally, 1,043 cases of esophageal cancer were diagnosed and histologically confirmed as squamous cell carcinoma by two pathologists independently. Patients with a history of any other types of cancer or with metastasis or those who had received neoadjuvant therapy were excluded. Around the same time, 1,143 noncancer patients from both hospitals were enrolled, with a frequency matching by age (±5 years) and gender, and the majority of them were admitted for trauma.

For 1,043 patients and 1,143 negative controls, baseline information such as age, gender, and other ESCC-related risk factors, such as smoking and drinking, were gathered through a questionnaire. A total of 1,143 control individuals and all case subjects provided feedback. Each participant had a 2 mL blood sample obtained intravenously for analysis.

The protocol adhered to the *Declaration of Helsinki* on the ethical conduct of research involving human/animal subjects and was approved by the Ethics Committee of Jiangsu University (Zhenjiang, China). All participants signed an informed consent form before recruitment.

### 2.2. Genomic DNA Extraction and Single-Nucleotide Polymorphism Analysis

The QIAamp DNA Blood Mini Kit was applied to amplify genomic DNA isolated from peripheral blood using PCR (Qiagen, Berlin, Germany). Samples were genotyped further using the ligation detection reaction (LDR) approach (supported by Genesky Biotechnology Inc., Shanghai, China). Six LAMA1 SNP loci (rs62081531, rs621993, rs539713, rs566655, rs73938538, and rs607230) were selected and analyzed. As a methodology of quality control, the analysis is repeated on 10% of randomly selected samples. In a preliminary study, we performed a linkage disequilibrium analysis on the 1000Genomes database, identified SNP loci with correlations, and further explored tag SNPs.

### 2.3. Surgical and Histological Evaluation

All patients underwent esophagectomy by qualified surgeons. Following the procedure, surgical specimens will be fixed to a cork and immersed in 10% formalin. All these patients' specimens were systematically reevaluated by experienced pathologists specialized in thoracic oncology and restaged under the 8th edition AJCC/UICC staging of cancers of the esophagus and esophagogastric junction. The histopathological examination includes tumor size, grade of differentiation, the margin of resection, and lymph node status.

### 2.4. Statistical Analysis

Statistical analyses were performed using SPSS version 26 (IBM, Chicago, IL) and *R* software (Version 4.0.3, *R* Foundation for Statistical Computing, Vienna, Austria). The baseline characteristics were summarized using the *R* software package “tableone”. Clinical characteristics were compared using *Fisher's* exact test or the *χ*^2^ test for categorical variables and the *Student's t*-test for comparing continuous variables. For the genetic model (A as a major allele and B as a minor allele), (a) *dominant model*: allele B increases risk; (b) *Recessive model*: two copies of minor allele B are required for increased risk; (c) *additive model*: r-fold increased risk for AB and 2r increased risk for BB; (d) *multiplicative model*: r-fold increased risk for AB and r2 increased risk for BB [16]. Two-tailed *p* value < 0.05 is considered as statistical significance, whereas *p* values between 0.05 and 0.10 are considered borderline statistically significant. The crude odd ratio (OR) and its corresponding 95% confidence interval (CI) are calculated according to genotypes between the two groups. In most cases, the risk is compared using the parametric test. When the sample size in the group is small, we use the nonparametric test. For stratified analyses, we included age, gender, smoking and drinking status, and the SNP model in the analysis, resulting in an adjusted OR for the SNP model. The adjusted OR and its corresponding CI are calculated by logistic regression analysis, and the hierarchical analysis is carried out. Demographic data, including age, sex, alcohol, and smoking, were covariates. The genotype was a dummy variable, and the group was a dependent variable. The SHEsis online platform [17] was utilized to conduct linkage disequilibrium studies and visualize the results by using *R* 4.0.3 and the *R* software packages “LDheatmap” and “genetics,” and SHEsis was also used to conduct haplotype frequency analyses [18].

## 3. Results

Between October 2008 and January 2017, we included 1,043 patients and 1,143 negative controls. There was no statistically significant difference in median age between the case and control groups [63.00 (59.00, 68.00) versus 63.00 (54.00, 70.00), *p*=0.257]. Women accounted for 27.3% of the case group and 27.6% of the control group (*P*=0.941). The proportion of smoking and drinking in ESCC patients was higher than that in the control group (43.5% vs. 29.7%, *P* < 0.001, 31.5% vs. 16.3%, *P* < 0.001). Detailed clinicopathological information was described in Table 1.

**Figure 1:**
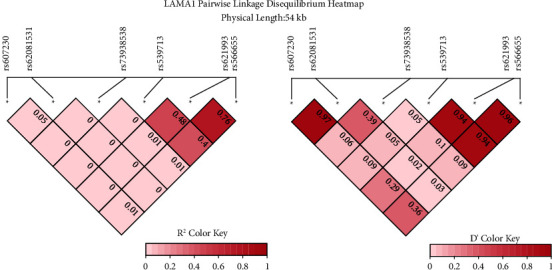
D′ (normalized D) or r2 (correlation coefficient) of linkage disequilibrium on 6 SNP loci of *LAMA1*.

The brief information on the six genotyped SNPs of *LAMA1* is shown in Table 2. All SNP genotyping experiments were successful at a rate greater than 95%. The control group's minor allele frequencies (MAF) were similar to those found in East Asian groups in the 1000 Genomes and gnomAD-Genomes databases. The Hardy–Weinberg equilibrium (HWE) test revealed that all six SNPs in the control group had *p* values greater than 0.05, indicating that the control group was genetically equilibrium. Linkage disequilibrium of 6 SNP loci of *LAMA1* is shown in Figure 1 and the coefficient of linkage disequilibrium and correlation coefficient test are described in Tables S7a and S7b.

The association of 6 SNPs in *LAMA1* with esophageal squamous cell carcinoma is shown in Table 3. In the rs62081531 locus, G/A was a protective factor for ESCC compared with G/G (OR: 0.820, 95% CI: 0.677–0.993, *P*=0.042). We can also find in the dominant model that the minor allele A is a protective factor for ESCC (OR: 0.830, 95% CI: 0.691–0.997, *P*=0.046). At rs607230, T/C was a protective factor for ESCC compared with T/T (OR: 0.613, 95% CI: 0.389–0.965, *P*=0.034).

Simultaneously, we performed stratified analyses on 6 loci of *LAMA1,* and detailed information is present in Tables S1–S6. In rs62081531 G > A, in the younger population, the dominant model (OR: 0.680, 95% CI: 0.527–0.877, *P*=0.003), the recessive model (OR: 0.741, 95% CI: 0.594–0.923, *P*=0.008), or the multiplicative model (OR: 0.754, *P*=0.010) all imply a negative link with ESCC incidence. In those without alcohol consumption, we found that either the dominant model (OR: 0.777, 95% CI: 0.627–0.963, *P*=0.022), the recessive model (OR: 0.796, 95% CI: 0.660–0.961, *P* = 0.018), or the multiplicative model (OR: 0.800, 95% CI: 0.664–0.964, *P* = 0.019) reveals that it is a protective factor for esophageal cancer (Table S1). At the rs539713 A > G locus, in the former or current drinking population, we found that in the recessive or multiplicative model, it was associated with a reduced risk of ESCC (OR: 0.443, 95% CI: 0.224–0.876, *P* = 0.019 and OR: 0.744, 95% CI: 0.556–0.996, *P*=0.047) (Table S3). At the rs566655 T > G locus, we discovered that both the additive model (OR: 1.270, 95% CI: 1.006–1.603, *P* = 0.044) and the multiplicative model (OR: 1.274, 95% CI: 1.007–1.611, *P* = 0.044) were linked with an elevated risk of ESCC in individuals younger than 65 years old (Table S4). It was found that the rs607230 T > C variant was associated with a decreased risk of esophageal cancer in adults aged 65 years (OR: 0.427, 95% CI: 0.196–0.930, *P* = 0.032) (Table S6). After stratified analysis, rs621993 G > A and rs73938538 A > C did not seem to be linked with esophageal cancer susceptibility (Table S2 and S5). We also analyzed the association of *LAMA1* 6 SNPs with tumor differentiation and lymph node metastasis, and the detailed information is shown in Table S8. We found that neither the lymph node positivity rate nor the degree of differentiation was significantly associated with the mutation status of the 6 loci. Haplotype frequencies analysis revealed that A_rs62081531_G_rs621993_A_rs539713_T_rs566655_A_rs73938538_C_rs607230_ (OR: 0.803, 95% CI: 0.660∼0.977, *P*=0.028) and G_rs62081531_G_rs621993_A_rs539713_T_rs566655_C_rs73938538_C_rs607230_ (OR: 0.679, 95% CI: 0.504∼0.913, *P* = 0.010) were associated with less susceptibility of ESCC (Table 4).

## 4. Discussion

Through our multicenter large-samplecase-control study, we found that rs62081531 locus G > A and rs607230 locus T > C of *LAMA1* were independent protective factors for esophageal squamous cell carcinoma, especially in those younger than 65 and nondrinkers.

Haplotype frequency analysis revealed that A_rs62081531_G_rs621993_A_rs539713_T_rs566655_A_rs73938538_C_rs607230_ and G_rs62081531_G_rs621993_A_rs539713_T_rs566655_C_rs73938538_C_rs607230_ were associated with less susceptibility to ESCC among the Chinese population. To our knowledge, this is the first report on *LAMA1* single-nucleotide polymorphisms and susceptibility to esophageal squamous cell carcinoma based on large-scale multicenter clinical research.

LAMA1 (laminin subunit *α*1) is a part of laminin, a glycoprotein found in the extracellular matrix that constitutes the basement membrane, and has been shown to be involved in the occurrence and development of various diseases [11]. Tissue distribution of laminin subunit *α*1 is mainly in early epithelial development and some adult epithelia [8]. In terms of carcinogenesis, laminin plays an essential role in cell adhesion, mitosis, differentiation, and even metastasis [19]. Laminin is a fundamental functional component of the basement membrane of several tissues, including the endothelium of the vessel wall, and different isoforms may contribute to vascular homeostasis [20]. The *α*1 subunit of laminin is typically confined to capillary walls and is expressed in the basal layer of capillaries in the central nervous system [21]. A recent study demonstrates that laminin-1 functions as a chemoattractant for both stromal and vascular cells, as well as in epithelial/stromal cell interactions for the structure of the basement membrane and segregation of integrins, hence signaling the proliferation of epithelial cells [13]. Similarly, in colorectal cancer, Wu et al. reported novel driver mutations occurring during adenoma and cancer evolution by single-cellwhole-exome sequencing (scWES), with *LAMA1* (PI3K-Akt signaling pathway) being one of the most critical pathways for CRC evolution [22]. Likewise, Gudjonsson and coworkers revealed that laminin-1 plays a vital role in the replacement of myoepithelial cells in polarity reversal in breast cancer [23]. LAMA1 (laminin *α*1) mutations are highly related to retinal avascularity and neovascularization in nontumor fields, such as the Poretti–Boltshauser syndrome [24]. Regardless of tumor or other nontumor diseases, LAMA1 is essential for vascular homeostasis and the basal layer of blood vessels. Interestingly, Velling et al. found that none of the colon cancer cell lines synthesized the laminin *α*1 protein, and they suggested that mutations in the *LAMA1* gene may underlie the lack of laminin *α*1 chains observed in some colon cancers [25]. In our study, *LAMA1* mutation showed protective factors in both rs62081531 G > A and rs607230 T > C, and basic research also found that *LAMA1* deficiency could inhibit the proliferation and invasion of esophageal cancer [26, 27]. Clearly, not all *LAMA1* SNPS are protective factors. People younger than 65 years of age indicate that the mutation at rs566655 T > G increases the risk of esophageal cancer, which may be correlated to the function of a certain SNP.

However, there are few reports on *LAMA1* and esophageal cancer. Most of the research is limited to the genetic function of *LAMA1*. Meng and colleagues found that laminin *α*1 (LAMA1) is highly expressed in ESCC tissue and mediates the FAK-PI3K-Akt signaling pathway [27]. Zhou et al. revealed that *LAMA1* was significantly upregulated in ESCC tissues and positively correlated with an aggressive oncogenic phenotype [26]. Nevertheless, the relationship between *LAMA1* SNP and disease in malignancies has not been demonstrated. Previous research has focused chiefly on nontumor studies such as chronic disease or degenerative disease. Zhao et al. showed that rs2089760 T > G, which is located in the *LAMA1* promoter region, may be associated with myopia in Chinese populations [28]. Similarly, the *LAMA1* rs2089760 G > A mutation was reported to reduce transcription factor binding ability and transcription initiation activity and negatively control the gene transcription of *LAMA1*, playing a crucial role in pathological myopia [29]. In a study on degenerative diseases, D'Aoust and colleagues were the first to discover that *LAMA1* rs73938538 A > C was positively related to Alzheimer's disease in the Amish community [30]. Due to the single-nucleotide polymorphism of *LAMA1*, the mutated site seems unable to effectively translate LAMA1 into laminin subunit *α*1 so as to exert its specific biological function, hence preventing esophageal cancer susceptibility. We observed that the consequences of two SNP loci that were related to esophageal susceptibility were stop-gain mutations, while the rest were synonymous and missense variants. This is largely in accordance with our expectations, especially when terminal gain mutations and missense variants can dramatically alter protein function, even with single nucleotide changes. However, we also reveal that in the ESCC population, *LAMA1* polymorphisms did not show a statistically significant association with the degree of differentiation, lymph node positivity, or T stage.

The main limitation of our study is that we only included populations from a specific region in eastern China, which may result in a certain geographical specificity and may not be generalized to the entire ESCC population. In addition, our study lacks replication in independent cohorts. Furthermore, we only discovered SNP sites in peripheral blood in our investigation. How these SNPs of *LAMA1* translate into biological function in the evolution of esophageal carcinoma is definitely a primary subject of our future investigation, which is currently technically difficult due to the lack of biological tools in our lab.

In conclusion, we found a strong association of *LAMA1* rs62081531, rs539713, rs566655, and rs607230 polymorphisms with esophageal cancer susceptibility in the Chinese population. *LAMA1* SNPs may significantly impact the occurrence of esophageal cancer and may serve as effective diagnostic biomarkers.

## Figures and Tables

**Table 1:** Distribution of clinicopathological characteristics in the ESCC case and control groups.

	Case	Control	*p* value
	(*n* = 1043)	(*n* = 1143)	
Age (median (IQR))	63.00 (59.00, 68.00)	63.00 (54.00, 70.00)	0.257
Gender (%)			
Female	285 (27.3)	315 (27.6)	0.941
Male	758 (72.7)	828 (72.4)	
Smoke status (%)			
No	589 (56.5)	803 (70.3)	<0.001
Former/current	454 (43.5)	340 (29.7)	
Alcohol consumption (%)			
No	714 (68.5)	957 (83.7)	<0.001
Former/current	329 (31.5)	186 (16.3)	
BMI (median (IQR))	22.27 (20.20, 24.35)	23.88 (21.89, 25.88)	<0.001
Chronic disease (%)			
No	797 (76.4)	604 (52.8)	<0.001
Yes	246 (23.6)	539 (47.2)	
Hypertension (%)	234 (22.4)	416 (36.4)	
Diabetes (%)	31 (3.0)	216 (18.9)	
Cardiovascular disease (%)	0 (0.0)	59 (5.2)	
pT stage (%)			
Tis	1 (0.1)		
T1a	33 (3.2)		
T1b	112 (10.7)		
T2	382 (36.6)		
T3	513 (49.2)		
T4	2 (0.2)		
pN stage (%)			
N0	775 (74.3)		
N1	201 (19.3)		
N2	45 (4.3)		
N3	22 (2.1)		
Differentiation (%)			
High	368 (35.3)		
Moderate	537 (51.5)		
Low	138 (13.2)		

Data are no. (%) or mean (SD) or median (IQR); BMI: body mass index; pT stage: pathological T stage; pN stage: pathological N stage.

**Table 2:** Primary information of LAMA1.

Gene	LAMA1					
Genotyped SNP	rs62081531	rs621993	rs539713	rs566655	rs73938538	rs607230
Allele	G > A	G > A	A > G	T > G	A > C	T > C
Consequence	Synonymous variant	Synonymous variant	Stop gained	Missense variant	Synonymous variant	Stop gained
RegulomeDB rank^†^	7	4	5	5	5	4
Chromosome	18					
Chromosome position^‡^	6986260	7033038	7017323	7034509	7008584	6980524
MAF in control	*A* = 0.1810	*A* = 0.1731	*G* = 0.2768	*G* = 0.1432	C = 0.1142	*T* = 0.1913
MAF in 1000 genomes						
Global	*A* = 0.1378	*A* = 0.4065	*G* = 0.5475	*G* = 0.1502	C = 0.1717	*T* = 0.3063
East Asian	*A* = 0.1915	*A* = 0.1319	*G* = 0.2262	*G* = 0.1042	C = 0.1091	*T* = 0.1756
MAF in gnomAD-genomes						
Global	*A* = 0.178903	*A* = 0.468719	*G* = 0.612173	*G* = 0.203225	C = 0.157585	*T* = 0.319456
East Asian	*A* = 0.1986	*A* = 0.1629	*G* = 0.2540	*G* = 0.1313	C = 0.1097	*T* = 0.1621
*p* value for the HWE test in the controls	0.9432	0.9743	0.9996	0.7212	0.8633	0.6770
Genotyping method	LDR					
% genotyping value	98.90%	98.95%	98.95%	98.95%	98.86%	98.03%

^†^ https://www.regulomedb.org/. ^‡^Based on Genome Reference Consortium Human Build 38(GRCh38).

**Table 3:** Association between LAMA1 single-nucleotide polymorphism and esophageal squamous cell carcinoma while controlling age and gender

Locus	Genotype	ESCC case	Control	Codominant model		Dominant model		Recessive model		Additive model		Multiplicative model	
				Or (95%CI)	*p*value	Or (95% CI)	*p*value	Or (95% CI)	*p*value	Or (95% CI)	*p*value	Or (95% CI)	*p*value
rs62081531	G/G	729 (69.9)	765 (66.9)	Ref		0.830 (0.691–0.997)	**0.046**	0.968 (0.606–1.545)	0.891	0.870 (0.743–1.017)	0.081	0.866 (0.739–1.015)	0.076
	G/A	261 (25.0)	334 (29.2)	0.820 (0.677–0.993)	**0.042**								
	A/A	34 (3.3)	39 (3.4)	0.915 (0.571–1.465)	0.711								

rs621993	G/G	704 (67.5)	777 (68.0)	Ref		0.981 (0.818–1.177)	0.839	1.114 (0.682–1.818)	0.666	0.997 (0.852–1.167)	0.970	0.997 (0.851–1.167)	0.970
	G/A	288 (27.6)	328 (28.7)	0.969 (0.803–1.17)	0.744								
	A/A	33 (3.2)	33 (2.9)	1.104 (0.674–1.807)	0.695								

rs539713	A/A	525 (50.3)	595 (52.1)	Ref		1.044 (0.881–1.236)	0.621	0.805 (0.576–1.124)	0.202	0.991 (0.866–1.135)	0.900	0.992 (0.868–1.133)	0.902
	G/A	436 (41.8)	456 (39.9)	1.084 (0.909–1.292)	0.371								
	G/G	64 (6.1)	87 (7.6)	0.834 (0.591–1.175)	0.299								

rs566655	T/T	738 (70.8)	832 (72.8)	Ref		1.057 (0.875–1.277)	0.563	1.340 (0.736–2.441)	0.338	1.071 (0.904–1.268)	0.430	1.070 (0.904–1.266)	0.432
	T/G	263 (25.2)	286 (25.0)	1.037 (0.853–1.259)	0.717								
	G/G	24 (2.3)	20 (1.7)	1.353 (0.741–2.469)	0.325								

rs73938538	A/A	829 (79.5)	891 (78.0)	Ref		0.844 (0.684–1.042)	0.115	0.854 (0.373–1.957)	0.710	0.857 (0.706–1.042)	0.122	0.859 (0.707–1.042)	0.124
	C/A	184 (17.6)	234 (20.5)	0.845 (0.682–1.048)	0.125								
	C/C	10 (1.0)	13 (1.1)	0.827 (0.361–1.896)	0.653								

rs607230	T/T	49 (4.7)	37 (3.2)	Ref		0.657 (0.425–1.016)	0.059	1.046 (0.875–1.25)	0.624	0.981 (0.844–1.140)	0.803	0.981 (0.842–1.142)	0.801
	T/C	293 (28.1)	361 (31.6)	0.613 (0.389–0.965)	**0.034**								
	C/C	664 (63.7)	739 (64.7)	0.678 (0.437–1.053)	0.084								

OR: odds ratio; CI: confidence interval.

**Table 4:** Haplotype frequencies in the case and control groups and risk of ESCC.

Haplotype (LAMA1)	Case (freq)	Control (freq)	Chi2	Or [95% CI]	*p* value
A_rs62081531_A_rs621993_G_rs539713_G_rs566655_A_rs73938538_C_rs607230_	38.73 (1.9%)	63.18 (2.8%)	—	—	
A_rs62081531_A_rs621993_G_rs539713_G_rs566655_C_rs73938538_C_rs607230_	4.99 (0.2%)	2.60 (0.1%)	—	—	
A_rs62081531_A_rs621993_G_rs539713_T_rs566655_A_rs73938538_C_rs607230_	2.33 (0.1%)	4.12 (0.2%)	—	—	
A_rs62081531_G_rs621993_A_rs539713_T_rs566655_A_rs73938538_C_rs607230_	200.31 (10.0%)	269.91 (11.9%)	4.823	0.803 [0.660∼0.977]	**0.028**
A_rs62081531_G_rs621993_A_rs539713_T_rs566655_C_rs73938538_C_rs607230_	13.40 (0.7%)	15.93 (0.7%)	—	—	
A_rs62081531_G_rs621993_G_rs539713_T_rs566655_A_rs73938538_C_rs607230_	38.33 (1.9%)	56.03 (2.5%)	—	—	
G_rs62081531_A_rs621993_A_rs539713_G_rs566655_A_rs73938538_C_rs607230_	7.10 (0.4%)	4.11 (0.2%)	—	—	
G_rs62081531_A_rs621993_A_rs539713_T_rs566655_A_rs73938538_C_rs607230_	1.29 (0.1%)	3.52 (0.2%)	—	—	
G_rs62081531_A_rs621993_G_rs539713_G_rs566655_A_rs73938538_C_rs607230_	168.31 (8.4%)	171.67 (7.6%)	0.731	1.102 [0.881∼1.379]	0.393
G_rs62081531_A_rs621993_G_rs539713_G_rs566655_A_rs73938538_T_rs607230_	18.19 (0.9%)	30.69 (1.4%)	—	—	
G_rs62081531_A_rs621993_G_rs539713_G_rs566655_C_rs73938538_C_rs607230_	29.83 (1.5%)	39.59 (1.7%)	—	—	
G_rs62081531_A_rs621993_G_rs539713_G_rs566655_C_rs73938538_T_rs607230_	3.90 (0.2%)	7.69 (0.3%)	—	—	
G_rs62081531_A_rs621993_G_rs539713_T_rs566655_A_rs73938538_C_rs607230_	29.88 (1.5%)	35.63 (1.6%)	—	—	
G_rs62081531_A_rs621993_G_rs539713_T_rs566655_A_rs73938538_T_rs607230_	12.55 (0.6%)	18.16 (0.8%)	—	—	
G_rs62081531_A_rs621993_G_rs539713_T_rs566655_C_rs73938538_C_rs607230_	6.78 (0.3%)	3.95 (0.2%)	—	—	
G_rs62081531_G_rs621993_A_rs539713_T_rs566655_A_rs73938538_C_rs607230_	845.42 (42.2%)	909.38 (40.1%)	1.066	1.072 [0.939∼1.224]	0.302
G_rs62081531_G_rs621993_A_rs539713_T_rs566655_A_rs73938538_T_rs607230_	259.49 (12.9%)	275 (11.9%)	0.720	1.083 [0.900∼1.303]	0.396
G_rs62081531_G_rs621993_A_rs539713_T_rs566655_C_rs73938538_C_rs607230_	74.63 (3.7%)	119.97 (5.3%)	6.626	0.679 [0.504∼0.913]	**0.010**
G_rs62081531_G_rs621993_A_rs539713_T_rs566655_C_rs73938538_T_rs607230_	40.47 (2.0%)	43.28 (1.9%)	—	—	
G_rs62081531_G_rs621993_G_rs539713_T_rs566655_A_rs73938538_C_rs607230_	117.31 (5.9%)	114.20 (5.0%)	1.127	1.155 [0.885∼1.508]	0.288
G_rs62081531_G_rs621993_G_rs539713_T_rs566655_A_rs73938538_T_rs607230_	34.02 (1.7%)	59.00 (2.6%)	—	—	
G_rs62081531_G_rs621993_G_rs539713_T_rs566655_C_rs73938538_C_rs607230_	8.56 (0.4%)	15.83 (0.7%)	—	—	

Freq: frequency; OR: odds ratio; CI: confidence interval; p < 0.05 is regarded as statistically significant value.

## Data Availability

Statistical results of this study are available from corresponding authors upon reasonable request. Specific patient clinical information and genetic data to support the study results have not yet been made available due to the National BioSafety Law of the People's Republic of China.

## Abbreviations

EC: Esophageal cancer
ESCC: Esophageal squamous cell carcinoma
SNP: Single-nucleotide polymorphism.

## Supplementary Materials

Supplementary Materials Table S1: Stratified analyses between rs62081531 G > A polymorphism and ESCC risk by age, gender, smoking status, and alcohol consumption. Table S2: Stratified analyses between rs621993 G > A polymorphism and ESCC risk by age, gender, smoking status, and alcohol consumption. Table S3: Stratified analyses between rs539713 A > G polymorphism and ESCC risk by age, gender, smoking status, and alcohol consumption. Table S4: Stratified analyses between rs566655 T > G polymorphism and ESCC risk by age, gender, smoking status, and alcohol consumption. Table S5: Stratified analyses between rs73938538 A > C polymorphism and ESCC risk by age, gender, smoking status, and alcohol consumption. Table S6: Stratified analyses between rs607230 T > C polymorphism and ESCC risk by age, gender, smoking status, and alcohol consumption. Table S7a: The linkage disequilibrium test of LAMA1 in the case and control groups. Table S7b: The linkage disequilibrium test of LAMA1 in the case and control groups. Table S8: The pathological baseline of 1043 ESCC patients on LAMA1 single-nucleotide polymorphism. pT stage: pathological T stage; pN stage: pathological N stage.
