# Supplementary material for: Association of LAMA1 Single-Nucleotide Polymorphisms with Risk of Esophageal Squamous Cell Carcinoma among the Eastern Chinese Population
Source: J Oncol. 2023 Feb 14;2023:6922909. doi: 10.1155/2023/6922909 (PMC9943613; doi:10.1155/2023/6922909)
Supplement: Supplementary Materials — Table S1: Stratified analyses between rs62081531 G > A polymorphism and ESCC risk by age, gender, smoking status, and alcohol consumption. Table S2: Stratified analyses between rs621993 G > A polymorphism and ESCC risk by age, gender, smoking status, and alcohol consumption. Table S3: Stratified analyses between rs539713 A > G polymorphism and ESCC risk by age, gender, smoking status, and alcohol consumption. Table S4: Stratified analyses between rs566655 T > G polymorphism and ESCC risk by age, gender, smoking status, and alcohol consumption. Table S5: Stratified analyses between rs73938538 A > C polymorphism and ESCC risk by age, gender, smoking status, and alcohol consumption. Table S6: Stratified analyses between rs607230 T > C polymorphism and ESCC risk by age, gender, smoking status, and alcohol consumption. Table S7a: The linkage disequilibrium test of LAMA1 in the case and control groups. Table S7b: The linkage disequilibrium test of LAMA1 in the case and control groups. Table S8: The pathological baseline of 1043 ESCC patients on LAMA1 single-nucleotide polymorphism. pT stage: pathological T stage; pN stage: pathological N stage. [file 6922909.f1.docx]

**Table S1 Stratified Analyses Between rs62081531 G>A Polymorphism and ESCC Risk by Age, Gender, Smoking Status, and Alcohol Consumption**

|  |  | **Case/Control** | | | **Dominant Model** | | **Recessive Model** | | **Additive Model** | | **Multiplicative Model** | |
| --- | --- | --- | --- | --- | --- | --- | --- | --- | --- | --- | --- | --- |
|  |  | **GG** | **GA** | **AA** | Adjusted OR (95%CI） | *P value* | Adjusted OR (95%CI） | *P value* | Adjusted OR (95%CI） | *P value* | Adjusted OR (95%CI） | *P value* |
|  |  | **729/765** | **261/334** | **34/39** |  |  |  |  |  |  |  |  |
| **Age** | ≥65 | 306/343 | 132/142 | 12/13 | 1.029 (0.778-1.362) | 0.839 | 1.104 (0.491-2.484) | 0.810 | 1.032 (0.808-1.317) | 0.803 | 1.032 (0.806-1.321) | 0.801 |
|  | <65 | 423/422 | 129/192 | 22/26 | 0.680 (0.527-0.877) | **0.003** | 0.907 (0.497-1.655) | 0.750 | 0.741 (0.594-0.923) | **0.008** | 0.754 (0.609-0.934) | **0.010** |
| **Gender** | Female | 197/206 | 77/100 | 6/8 | 0.829 (0.585-1.176) | 0.294 | 0.847 (0.288-2.488) | 0.762 | 0.855 (0.63-1.161) | 0.316 | 0.849 (0.621-1.161) | 0.305 |
|  | Male | 532/559 | 184/234 | 28/31 | 0.820 (0.656-1.025) | 0.081 | 1.010 (0.589-1.733) | 0.971 | 0.863 (0.712-1.047) | 0.135 | 0.870 (0.721-1.050) | 0.146 |
| **Smoke** | No | 406/535 | 151/233 | 16/31 | 0.825 (0.652-1.042) | 0.107 | 0.763 (0.412-1.416) | 0.392 | 0.84 (0.685-1.029) | 0.092 | 0.843 (0.689-1.031) | 0.096 |
|  | Former/Current | 323/230 | 110/101 | 18/8 | 0.802 (0.585-1.098) | 0.169 | 1.507 (0.635-3.575) | 0.352 | 0.884 (0.672-1.162) | 0.377 | 0.887 (0.678-1.161) | 0.384 |
| **Alcohol** | No | 509/641 | 175/276 | 18/36 | 0.777 (0.627-0.963) | **0.022** | 0.717 (0.402-1.277) | 0.259 | 0.796 (0.660-0.961) | **0.018** | 0.800 (0.664-0.964) | **0.019** |
|  | Former/Current | 220/124 | 86/58 | 16/3 | 0.944 (0.639-1.395) | 0.773 | 2.936 (0.839-10.277) | 0.092 | 1.064 (0.759-1.493) | 0.718 | 1.062 (0.761-1.483) | 0.722 |

**Table S2 Stratified Analyses Between rs621993 G>A Polymorphism and ESCC Risk by Age, Gender, Smoking Status, and Alcohol Consumption**

|  |  | **Case/Control** | | | **Dominant Model** | | **Recessive Model** | | **Additive Model** | | **Multiplicative Model** | |
| --- | --- | --- | --- | --- | --- | --- | --- | --- | --- | --- | --- | --- |
|  |  | **GG** | **GA** | **AA** | Adjusted OR (95%CI） | *P value* | Adjusted OR (95%CI） | *P value* | Adjusted OR (95%CI） | *P value* | Adjusted OR (95%CI） | *P value* |
|  |  | **704/777** | **288/328** | **33/33** |  |  |  |  |  |  |  |  |
| **Age** | ≥65 | 313/328 | 125/154 | 12/16 | 0.903 (0.683-1.193) | 0.472 | 0.851 (0.392-1.849) | 0.684 | 0.912 (0.716-1.161) | 0.453 | 0.910 (0.713-1.162) | 0.450 |
|  | <65 | 391/449 | 163/174 | 21/17 | 1.100 (0.856-1.413) | 0.456 | 1.338 (0.686-2.610) | 0.392 | 1.109 (0.892-1.379) | 0.353 | 1.107 (0.892-1.374) | 0.357 |
| **Gender** | Female | 192/220 | 79/87 | 9/7 | 1.052 (0.739-1.498) | 0.778 | 1.411 (0.507-3.928) | 0.509 | 1.073 (0.788-1.462) | 0.654 | 1.074 (0.787-1.465) | 0.653 |
|  | Male | 512/557 | 209/241 | 24/26 | 0.994 (0.798-1.238) | 0.956 | 1.014 (0.567-1.813) | 0.963 | 0.997 (0.824-1.205) | 0.974 | 0.997 (0.825-1.205) | 0.974 |
| **Smoke** | No | 386/546 | 172/228 | 16/25 | 1.059 (0.841-1.335) | 0.624 | 0.892 (0.470-1.692) | 0.726 | 1.033 (0.845-1.262) | 0.754 | 1.033 (0.845-1.263) | 0.753 |
|  | Former/Current | 318/231 | 116/100 | 17/8 | 0.893 (0.653-1.221) | 0.478 | 1.565 (0.656-3.737) | 0.313 | 0.962 (0.732-1.263) | 0.778 | 0.962 (0.735-1.260) | 0.780 |
| **Alcohol** | No | 471/651 | 209/276 | 23/26 | 1.072 (0.870-1.322) | 0.513 | 1.219 (0.687-2.161) | 0.499 | 1.075 (0.897-1.289) | 0.435 | 1.076 (0.896-1.291) | 0.432 |
|  | Former/Current | 233/126 | 79/52 | 10/7 | 0.802 (0.539-1.195) | 0.278 | 0.831 (0.307-2.247) | 0.715 | 0.828 (0.587-1.170) | 0.284 | 0.836 (0.597-1.171) | 0.297 |

**Table S3 Stratified Analyses Between** **rs539713 A>G Polymorphism and ESCC Risk by Age, Gender, Smoking Status, and Alcohol Consumption**

|  |  | **Case/Control** | | | **Dominant Model** | | **Recessive Model** | | **Additive Model** | | | **Multiplicative Model** | | | |
| --- | --- | --- | --- | --- | --- | --- | --- | --- | --- | --- | --- | --- | --- | --- | --- |
|  |  | AA | AG | GG | Adjusted OR (95%CI） | *P value* | Adjusted OR (95%CI） | *P value* | Adjusted OR (95%CI） | *P value* | | Adjusted OR (95%CI） | *P value* | |  |
|  |  | 525/595 | 436/456 | 64/87 |  |  |  |  |  |  |  |  |  |  |  |
| Age | ≥65 | 234/255 | 187/210 | 29/33 | 0.995 (0.767-1.291) | 0.972 | 1.014 (0.600-1.715) | 0.958 | 0.999 (0.813-1.228) | | 0.994 | 0.999 (0.809-1.234) | | 0.994 |  |
|  | <65 | 291/340 | 249/246 | 35/54 | 1.064 (0.844-1.342) | 0.601 | 0.643 (0.407-1.014) | 0.057 | 0.966 (0.804-1.160) | | 0.708 | 0.965 (0.802-1.161) | | 0.706 |  |
| Gender | Female | 145/174 | 116/117 | 19/23 | 1.114 (0.803-1.547) | 0.517 | 0.884 (0.464-1.687) | 0.709 | 1.050 (0.809-1.363) | | 0.715 | 1.050 (0.808-1.365) | | 0.714 |  |
|  | Male | 380/421 | 320/339 | 45/64 | 1.007 (0.821-1.236) | 0.944 | 0.730 (0.485-1.097) | 0.130 | 0.956 (0.813-1.123) | | 0.580 | 0.954 (0.809-1.124) | | 0.572 |  |
| Smoke | No | 292/428 | 249/311 | 33/60 | 1.123 (0.904-1.394) | 0.294 | 0.745 (0.479-1.159) | 0.192 | 1.027 (0.865-1.219) | | 0.758 | 1.028 (0.864-1.224) | | 0.754 |  |
|  | Former/Current | 233/167 | 187/145 | 31/27 | 0.879 (0.658-1.175) | 0.385 | 0.810 (0.467-1.406) | 0.453 | 0.892 (0.711-1.119) | | 0.323 | 0.888 (0.705-1.119) | | 0.314 |  |
| Alcohol | No | 354/507 | 302/379 | 47/67 | 1.129 (0.927-1.373) | 0.227 | 0.936 (0.635-1.380) | 0.738 | 1.067 (0.914-1.246) | | 0.411 | 1.069 (0.914-1.252) | | 0.404 |  |
|  | Former/Current | 171/88 | 134/77 | 17/20 | 0.790 (0.547-1.141) | 0.209 | 0.443 (0.224-0.876) | **0.019** | 0.753 (0.566-1.000) | | **0.050** | 0.744 (0.556-0.996) | | **0.047** |  |

**Table S4 Stratified Analyses Between rs566655 T>G Polymorphism and ESCC Risk by Age, Gender, Smoking Status, and Alcohol Consumption**

|  |  | **Case/Control** | | | **Dominant Model** | | **Recessive Model** | | **Additive Model** | | **Multiplicative Model** | |
| --- | --- | --- | --- | --- | --- | --- | --- | --- | --- | --- | --- | --- |
|  |  | TT | TG | GG | Adjusted OR (95%CI） | *P value* | Adjusted OR (95%CI） | *P value* | Adjusted OR (95%CI） | *P value* | Adjusted OR (95%CI） | *P value* |
|  |  | 738/832 | 263/286 | 24/20 |  |  |  |  |  |  |  |  |
| Age | ≥65 | 332/350 | 110/136 | 8/12 | 0.903 (0.674-1.209) | 0.492 | 0.727 (0.289-1.828) | 0.498 | 0.900 (0.695-1.165) | 0.424 | 0.898 (0.692-1.166) | 0.419 |
|  | <65 | 406/482 | 153/150 | 16/8 | 1.255 (0.967-1.630) | 0.088 | 2.155 (0.896-5.182) | 0.086 | 1.270 (1.006-1.603) | **0.044** | 1.274 (1.007-1.611) | **0.044** |
| Gender | Female | 198/233 | 76/76 | 6/5 | 1.183 (0.822-1.703) | 0.366 | 1.405 (0.421-4.688) | 0.581 | 1.172 (0.847-1.621) | 0.339 | 1.176 (0.846-1.634) | 0.334 |
|  | Male | 540/599 | 187/210 | 18/15 | 1.063 (0.846-1.337) | 0.600 | 1.277 (0.626-2.607) | 0.502 | 1.071 (0.874-1.313) | 0.510 | 1.071 (0.873-1.314) | 0.508 |
| Smoke | No | 399/584 | 164/199 | 11/16 | 1.194 (0.940-1.516) | 0.145 | 0.924 (0.423-2.016) | 0.842 | 1.143 (0.925-1.412) | 0.216 | 1.147 (0.926-1.422) | 0.210 |
|  | Former/Current | 339/248 | 99/87 | 13/4 | 0.869 (0.624-1.209) | 0.404 | 2.506 (0.793-7.917) | 0.117 | 0.961 (0.715-1.291) | 0.791 | 0.962 (0.717-1.289) | 0.793 |
| Alcohol | No | 493/698 | 195/239 | 15/16 | 1.181 (0.950-1.467) | 0.134 | 1.271 (0.622-2.597) | 0.511 | 1.160 (0.957-1.407) | 0.131 | 1.166 (0.958-1.419) | 0.125 |
|  | Former/Current | 245/134 | 68/47 | 9/4 | 0.810 (0.534-1.229) | 0.321 | 1.409 (0.420-4.726) | 0.579 | 0.877 (0.606-1.268) | 0.485 | 0.882 (0.616-1.265) | 0.496 |

**Table S5 Stratified Analyses Between rs73938538 A>C Polymorphism and ESCC Risk by Age, Gender, Smoking Status, and Alcohol Consumption**

|  |  | **Case/Control** | | | **Dominant Model** | | **Recessive Model** | | **Additive Model** | | **Multiplicative Model** | |
| --- | --- | --- | --- | --- | --- | --- | --- | --- | --- | --- | --- | --- |
|  |  | AA | AC | CC | Adjusted OR (95%CI） | *P value* | Adjusted OR (95%CI） | *P value* | Adjusted OR (95%CI） | *P value* | Adjusted OR (95%CI） | *P value* |
|  |  | 829/891 | 184/234 | 10/13 |  |  |  |  |  |  |  |  |
| Age | ≥65 | 367/390 | 78/102 | 4/6 | 0.820 (0.591-1.138) | 0.235 | 0.708 (0.194-2.577) | 0.600 | 0.829 (0.613-1.121) | 0.223 | 0.828 (0.612-1.121) | 0.222 |
|  | <65 | 462/501 | 106/132 | 6/7 | 0.868 (0.652-1.156) | 0.334 | 1.041 (0.338-3.202) | 0.944 | 0.889 (0.683-1.158) | 0.383 | 0.888 (0.681-1.158) | 0.380 |
| Gender | Female | 223/252 | 55/57 | 1/5 | 1.051 (0.699-1.580) | 0.812 | 0.206 (0.024-1.790) | 0.152 | 0.972 (0.667-1.417) | 0.883 | 0.972 (0.666-1.419) | 0.882 |
|  | Male | 606/639 | 129/177 | 9/8 | 0.784 (0.608-1.010) | 0.060 | 1.359 (0.510-3.626) | 0.540 | 0.828 (0.655-1.046) | 0.113 | 0.826 (0.653-1.045) | 0.112 |
| Smoke | No | 467/622 | 101/164 | 4/13 | 0.791 (0.603-1.037) | 0.090 | 0.419 (0.135-1.300) | 0.132 | 0.781 (0.608-1.002) | 0.052 | 0.781 (0.608-1.003) | 0.053 |
|  | Former/Current | 362/269 | 83/70 | 6/0 | 0.947 (0.661-1.355) | 0.764 | / | / | 1.029 (0.737-1.436) | 0.866 | 1.030 (0.733-1.447) | 0.864 |
| Alcohol | No | 570/745 | 125/197 | 7/11 | 0.833 (0.652-1.064) | 0.143 | 0.884 (0.339-2.304) | 0.801 | 0.851 (0.679-1.066) | 0.160 | 0.849 (0.677-1.066) | 0.158 |
|  | Former/Current | 259/146 | 59/37 | 3/2 | 0.891 (0.566-1.403) | 0.619 | 1.065 (0.169-6.695) | 0.946 | 0.910 (0.599-1.382) | 0.658 | 0.909 (0.596-1.385) | 0.656 |

**Table S6 Stratified Analyses Between rs607230 T>C Polymorphism and ESCC Risk by Age, Gender, Smoking Status, and Alcohol Consumption**

|  |  | **Case/Control** | | | **Dominant Model** | | **Recessive Model** | | **Additive Model** | | **Multiplicative Model** | |
| --- | --- | --- | --- | --- | --- | --- | --- | --- | --- | --- | --- | --- |
|  |  | TT | TC | CC | Adjusted OR (95%CI） | *P value* | Adjusted OR (95%CI） | *P value* | Adjusted OR (95%CI） | *P value* | Adjusted OR (95%CI） | *P value* |
|  |  | 49/37 | 293/361 | 664/739 |  |  |  |  |  |  |  |  |
| Age | ≥65 | 21/10 | 117/167 | 309/321 | 0.427 (0.196-0.930) | **0.032** | 1.185 (0.898-1.564) | 0.229 | 1.040 (0.819-1.320) | 0.748 | 1.040 (0.819-1.321) | 0.747 |
|  | <65 | 28/27 | 176/194 | 355/418 | 0.879 (0.504-1.535) | 0.651 | 0.883 (0.692-1.127) | 0.319 | 0.899 (0.732-1.105) | 0.313 | 0.903 (0.737-1.105) | 0.322 |
| Gender | Female | 11/10 | 78/103 | 186/201 | 0.750 (0.312-1.806) | 0.521 | 1.135 (0.803-1.605) | 0.473 | 1.062 (0.790-1.428) | 0.690 | 1.062 (0.790-1.429) | 0.690 |
|  | Male | 38/27 | 215/258 | 478/538 | 0.653 (0.388-1.098) | 0.108 | 0.957 (0.771-1.188) | 0.691 | 0.918 (0.764-1.103) | 0.360 | 0.920 (0.767-1.103) | 0.366 |
| Smoke | No | 24/28 | 176/259 | 362/512 | 0.803 (0.459-1.407) | 0.443 | 1.002 (0.798-1.256) | 0.989 | 0.976 (0.805-1.183) | 0.803 | 0.976 (0.804-1.184) | 0.802 |
|  | Former/Current | 25/9 | 117/102 | 302/227 | 0.493 (0.224-1.084) | 0.079 | 1.017 (0.746-1.385) | 0.917 | 0.925 (0.710-1.206) | 0.566 | 0.929 (0.718-1.203) | 0.577 |
| Alcohol | No | 31/29 | 210/309 | 448/614 | 0.652 (0.388-1.096) | 0.106 | 1.010 (0.822-1.242) | 0.921 | 0.960 (0.805-1.144) | 0.645 | 0.959 (0.804-1.145) | 0.644 |
|  | Former/Current | 18/8 | 83/52 | 216/125 | 0.744 (0.315-1.761) | 0.501 | 1.007 (0.680-1.492) | 0.972 | 0.960 (0.687-1.340) | 0.809 | 0.964 (0.702-1.323) | 0.819 |

**Table S7a Linkage Disequilibrium Test of LAMA1 in the Case and Control Group**

| D' | rs621993 | rs539713 | rs566655 | rs73938538 | rs607230 |
| --- | --- | --- | --- | --- | --- |
| rs62081531 | 0.016 | 0.053 | 0.030 | 0.388 | 0.965 |
| rs621993 | - | 0.940 | 0.957 | 0.100 | 0.287 |
| rs539713 | - | - | 0.944 | 0.047 | 0.089 |
| rs566655 | - | - | - | 0.086 | 0.357 |
| rs73938538 | - | - | - | - | 0.055 |

**Table S7b Linkage Disequilibrium Test of LAMA1 in the Case and Control Group**

| r^2^ | rs621993 | rs539713 | rs566655 | rs73938538 | rs607230 |
| --- | --- | --- | --- | --- | --- |
| rs62081531 | 0 | 0.002 | 0.001 | 0.004 | 0.046 |
| rs621993 | - | 0.484 | 0.756 | 0.006 | 0.004 |
| rs539713 | - | - | 0.404 | 0.001 | 0.001 |
| rs566655 | - | - | - | 0.005 | 0.005 |
| rs73938538 | - | - | - | - | 0.002 |

**Table S8 Pathological Baseline of 1043 ESCC patients on LAMA1 single nucleotide polymorphism**

|  | **rs62081531** | | | | **rs621993** | | | | **rs539713** | | | | **rs566655** | | | | **rs73938538** | | | | **rs607230** | | | |
| --- | --- | --- | --- | --- | --- | --- | --- | --- | --- | --- | --- | --- | --- | --- | --- | --- | --- | --- | --- | --- | --- | --- | --- | --- |
|  | A/A | G/A | G/G | *P value* | G/G | G/A | A/A | *P value* | A/A | G/A | G/G | *P value* | G/G | T/G | T/T | *P value* | A/A | C/A | C/C | *P value* | C/C | T/C | T/T | *P value* |
|  | 34 | 261 | 729 |  | 704 | 288 | 33 |  | 525 | 436 | 64 |  | 24 | 263 | 738 |  | 829 | 184 | 10 |  | 664 | 293 | 49 |  |
| **pT stage (%)** |  |  |  |  |  |  |  |  |  |  |  |  |  |  |  |  |  |  |  |  |  |  |  |  |
| T1a | 0 (0.0) | 9 (3.4) | 24 (3.4) | 0.996 | 23 (3.2) | 8 (2.8) | 3 (9.1) | 0.821 | 20 (3.8) | 10 (2.3) | 4 (6.2) | 0.898 | 1 (4.2) | 9 (3.4) | 24 (3.2) | 0.941 | 25 (3.0) | 9 (4.9) | 0 (0.0) | 0.967 | 22 (3.3) | 10 (3.4) | 0 (0.0) | 0.897 |
| T1b | 3 (8.8) | 30 (11.5) | 76 (10.4) |  | 71 (10.1) | 36 (12.5) | 2 (6.1) |  | 54 (10.3) | 48 (11.0) | 7 (10.9) |  | 2 (8.3) | 29 (11.0) | 78 (10.6) |  | 90 (10.9) | 18 (9.8) | 1 (10.0) |  | 76 (11.4) | 29 (9.9) | 3 (6.1) |  |
| T2 | 14 (41.2) | 92 (35.2) | 268 (36.8) |  | 253 (35.9) | 107 (37.2) | 14 (42.4) |  | 188 (35.8) | 164 (37.6) | 22 (34.4) |  | 13 (54.2) | 89 (33.8) | 272 (36.9) |  | 300 (36.2) | 70 (38.0) | 3 (30.0) |  | 241 (36.3) | 107 (36.5) | 22 (44.9) |  |
| T3 | 17 (50.0) | 129 (49.4) | 359 (49.2) |  | 355 (50.4) | 137 (47.6) | 14 (42.4) |  | 261 (49.7) | 214 (49.1) | 31 (48.4) |  | 8 (33.3) | 136 (51.7) | 362 (49.1) |  | 413 (49.8) | 86 (46.7) | 6 (60.0) |  | 323 (48.6) | 147 (50.2) | 24 (49.0) |  |
| T4 | 0 (0.0) | 1 (0.4) | 1 (0.1) |  | 2 (0.3) | 0 (0.0) | 0 (0.0) |  | 2 (0.4) | 0 (0.0) | 0 (0.0) |  | 0 (0.0) | 0 (0.0) | 2 (0.3) |  | 1 (0.1) | 1 (0.5) | 0 (0.0) |  | 2 (0.3) | 0 (0.0) | 0 (0.0) |  |
| **pN stage (%)** |  |  |  |  |  |  |  |  |  |  |  |  |  |  |  |  |  |  |  |  |  |  |  |  |
| N0 | 24 (70.6) | 198 (75.9) | 540 (74.1) | 0.294 | 514 (73.0) | 224 (77.8) | 25 (75.8) | 0.829 | 386 (73.5) | 329 (75.5) | 48 (75.0) | 0.743 | 17 (70.8) | 203 (77.2) | 543 (73.6) | 0.909 | 616 (74.3) | 138 (75.0) | 7 (70.0) | 0.698 | 486 (73.2) | 225 (76.8) | 38 (77.6) | 0.776 |
| N1 | 6 (17.6) | 44 (16.9) | 146 (20.0) |  | 143 (20.3) | 47 (16.3) | 6 (18.2) |  | 104 (19.8) | 81 (18.6) | 11 (17.2) |  | 6 (25.0) | 46 (17.5) | 144 (19.5) |  | 159 (19.2) | 34 (18.5) | 3 (30.0) |  | 137 (20.6) | 48 (16.4) | 8 (16.3) |  |
| N2 | 4 (11.8) | 15 (5.7) | 25 (3.4) |  | 30 (4.3) | 12 (4.2) | 2 (6.1) |  | 21 (4.0) | 18 (4.1) | 5 (7.8) |  | 1 (4.2) | 10 (3.8) | 33 (4.5) |  | 39 (4.7) | 5 (2.7) | 0 (0.0) |  | 27 (4.1) | 15 (5.1) | 2 (4.1) |  |
| N3 | 0 (0.0) | 4 (1.5) | 18 (2.5) |  | 17 (2.4) | 5 (1.7) | 0 (0.0) |  | 14 (2.7) | 8 (1.8) | 0 (0.0) |  | 0 (0.0) | 4 (1.5) | 18 (2.4) |  | 15 (1.8) | 7 (3.8) | 0 (0.0) |  | 14 (2.1) | 5 (1.7) | 1 (2.0) |  |
| **Differentiation (%)** |  |  |  |  |  |  |  |  |  |  |  |  |  |  |  |  |  |  |  |  |  |  |  |  |
| High | 9 (26.5) | 91 (34.9) | 261 (35.8) | 0.723 | 243 (34.5) | 110 (38.2) | 9 (27.3) | 0.605 | 183 (34.9) | 158 (36.2) | 21 (32.8) | 0.728 | 4 (16.7) | 98 (37.3) | 260 (35.2) | 0.378 | 286 (34.5) | 73 (39.7) | 3 (30.0) | 0.518 | 237 (35.7) | 92 (31.4) | 23 (46.9) | 0.299 |
| Moderate | 18 (52.9) | 135 (51.7) | 376 (51.6) |  | 367 (52.1) | 141 (49.0) | 21 (63.6) |  | 278 (53.0) | 215 (49.3) | 36 (56.2) |  | 17 (70.8) | 128 (48.7) | 384 (52.0) |  | 435 (52.5) | 88 (47.8) | 4 (40.0) |  | 337 (50.8) | 161 (54.9) | 23 (46.9) |  |
| Low | 7 (20.6) | 35 (13.4) | 92 (12.6) |  | 94 (13.4) | 37 (12.8) | 3 (9.1) |  | 64 (12.2) | 63 (14.4) | 7 (10.9) |  | 3 (12.5) | 37 (14.1) | 94 (12.7) |  | 108 (13.0) | 23 (12.5) | 3 (30.0) |  | 90 (13.6) | 40 (13.7) | 3 (6.1) |  |

pT stage: pathological T stage; pN stage: pathological N stage
